# Targeting pyroptosis in periodontitis: mechanisms and therapeutic strategies

**DOI:** 10.3389/fimmu.2026.1761693

**Published:** 2026-02-05

**Authors:** Yusen Qie, Jiaxin Li, Yang Xu, Sijia Liu, Na Liu, Qing Liu

**Affiliations:** 1 Hebei Key Laboratory of Stomatology, School and Hospital of Stomatology, Hebei Medical University, Shijiazhuang, Hebei, China; 2 Hebei Technology Innovation Center of Oral Health, School and Hospital of Stomatology, Hebei Medical University, Shijiazhuang, Hebei, China; 3 Department of Preventive Dentistry, School and Hospital of Stomatology, Hebei Medical University, Shijiazhuang, Hebei, China

**Keywords:** immune response, inflammasome, periodontal treatment, periodontitis, programmed cell death, pyroptosis

## Abstract

Periodontitis is a common chronic inflammatory disease of the oral cavity. It is characterized by progressive destruction of the periodontal supporting tissues and alveolar bone loss, and it remains a leading cause of tooth loss in adults. In recent years, pyroptosis, a pro-inflammatory form of programmed cell death, has attracted increasing attention for its role in the initiation and progression of periodontitis. This review summarizes the molecular mechanisms and signaling pathways of pyroptosis. We highlight pyroptosis-targeted therapeutic strategies for periodontitis, including molecular targeted interventions, gene regulation, ion channel blockers, and natural compounds, among others. We also discuss major challenges and translational prospects in light of recent advances. This review aims to provide direction and guidance for developing novel periodontal therapeutic and regenerative strategies by targeting pyroptosis.

## 1 Introduction

Periodontitis is a highly prevalent chronic infectious-inflammatory disease that affects more than one billion people worldwide. It is characterized by persistent gingival inflammation, periodontal pocket formation and progressive alveolar bone loss. These changes can ultimately lead to tooth loss. The pathogenesis of periodontitis is multifactorial and involves an imbalance between microbial infection and the host immune response (1). Pyroptosis is a pro-inflammatory form of programmed cell death that has gained increasing recognition in recent years. It plays a critical role in defending against exogenous pathogen invasion and sensing endogenous danger signals (2). In periodontitis, lipopolysaccharide (LPS) and other virulence factors, which are derived from periodontal pathogens, can activate pyroptosis-associated inflammasomes in both host immune cells and periodontal tissue cells. Inflammasome activation promotes caspase-1 activation and gasdermin D (GSDMD) cleavage, leading to plasma membrane pore formation and inflammatory cytokine release. These inflammatory mediators promote collagen degradation and alveolar bone resorption, culminating in destruction of periodontal soft and hard tissues (2–5). Accordingly, targeting key nodes in the pyroptosis pathway may offer novel therapeutic strategies for the prevention and treatment of periodontitis.

## 2 Overview and features of pyroptosis

Pyroptosis is a form of programmed inflammatory cell death mediated by the gasdermin family, and it plays a critical role in host defense and disease pathogenesis. This mode of cell death was first described in the 1980s and 1990s, when pathogen-infected macrophages were found to undergo a caspase-1–dependent form of cell death that was distinct from classical apoptosis (6, 7). Apoptotic cells typically maintain membrane integrity, whereas pyroptotic cells lose plasma membrane integrity and release intracellular contents. A major breakthrough occurred in 2015, when gasdermin D was identified as a key executioner of pyroptosis (8). Pyroptosis has distinct morphological and biological features. Early during pyroptosis, cells exhibit swelling and bubble-like protrusions, followed by plasma membrane rupture and the release of pro-inflammatory mediators. These mediators act as danger signals that activate immune responses in neighboring cells and amplify inflammation (9).

## 3 Molecular mechanisms of pyroptosis

**Figure 1:**
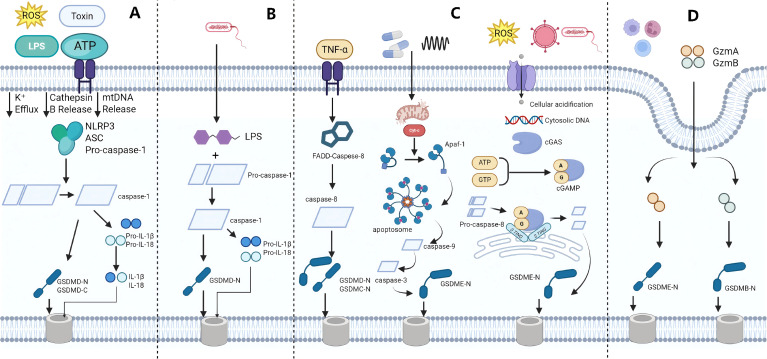
Molecular mechanisms of pyroptosis. **(A)** The canonical pathway. **(B)** Non-canonical pathway. **(C)** Other pathways. **(D)** Granzyme pathway. This diagram was created with BioRender.com.

Pyroptosis is primarily mediated by the gasdermin protein family. Its activation depends on GSDMD cleavage that liberates an N-terminal domain (GSDMD-N), which inserts into the plasma membrane to form pores and ultimately causes membrane rupture (10) (**Figure 1**). Two major molecular pathways have been described: the canonical pathway and non-canonical pathway. In the canonical pathway (**Figure 1A**), inflammasomes (e.g., the NLRP3 inflammasome) activate caspase-1, which cleaves GSDMD to generate the N-terminal fragment. GSDMD-N forms plasma membrane pores, causing cell swelling and lysis, promoting interleukin-1β (IL-1β) and interleukin-18 (IL-18) maturation, and amplifying inflammatory signaling (11–14). In the non-canonical pathway (**Figure 1B**), LPS directly activates caspase-4/5 or caspase-11. These caspases also cleave GSDMD to trigger pyroptosis. Although this pathway does not directly drive IL-1β and IL-18 maturation, the precursor forms of these cytokines can still be released through GSDMD pores and subsequently processed by extracellular proteases, which can provoke strong local and even systemic inflammation (15, 16).

Recent studies have identified additional pathways that can activate pyroptosis (**Figure 1C**). For example, in tumor immunity, caspase-8 can cleave GSDMD and GSDMC, which initiates pyroptotic signaling (17). caspase-3 can cleave GSDME, converting chemotherapy-induced apoptosis into pyroptosis and enhancing antitumor immune responses (18, 19). cGAS is a cytosolic DNA-sensing cyclic nucleotide synthase that activates the STING pathway by producing cGAMP. It plays key roles in antiviral immunity, inflammatory responses, and pyroptosis. The combination of cGAS activation and intracellular acidification can induce pyroptosis via the non-canonical cGAS-STING pathway. When cGAS is activated and intracellular pH decreases, STING forms aggregates on the endoplasmic reticulum, creating a platform for caspase-8-mediated GSDME cleavage and triggering pyroptosis (20). In addition, granzymes and pathogen effector proteins can directly cleave gasdermins to induce pyroptosis (21)(**Figure 1D**).

## 4 Association between pyroptosis and periodontitis

### 4.1 Periodontal pathogens can induce pyroptosis

Multiple periodontal pathogens have been shown to induce pyroptosis in host cells. As a key periodontal pathogen, *P. gingivalis* activates the NLRP3 inflammasome through its LPS and other virulence factors and promotes caspase-1-dependent GSDMD cleavage and IL-1β release (22–24). *P. gingivalis* infection can regulate macrophage pyroptosis via a glycolysis-related AMPK/SIRT/NF-κB signaling axis, exacerbating periodontal inflammation (25). Its outer membrane vesicles can also shift macrophage metabolism toward glycolysis and trigger inflammasome activation and pyroptosis (26). Other pathogens, such as *T. forsythia* and *T. denticola*, can also activate inflammasome pathways and induce pyroptosis in host cells (27–29).

### 4.2 Inflammasomes are activated in periodontitis

Inflammasomes serve as key molecular platforms that initiate pyroptosis (30). Among these, the NLRP3 inflammasome is the most extensively studied in periodontitis. The NLRP3 inflammasome comprises the pattern-recognition receptor NLRP3, the adaptor ASC, and the effector pro-caspase-1. In periodontitis, pathogens and their metabolites can activate the NLRP3 inflammasome, triggering caspase-1 activation and GSDMD-mediated pyroptosis (31–34). In diabetes-associated periodontitis, hyperglycemia impairs macrophage autophagic flux, promoting mitochondrial reactive oxygen species (mtROS) accumulation and NLRP3 inflammasome activation, worsening periodontal tissue destruction in rats (35). A related study showed that a hyperglycemic microenvironment synergizes with periodontal pathogens to drive caspase-1 activation and GSDMD cleavage, accelerating fibroblast senescence and periodontal tissue destruction. NLRC4 phosphorylation was identified as a key node in this process, and inhibiting this pathway markedly reduced pyroptosis and inflammatory responses (36). Chen et al. generated NLRP3-deficient mice and established a ligature-induced periodontitis model. NLRP3 deficiency significantly reduced osteoclast precursor numbers, osteoclast differentiation, and alveolar bone loss in this model. Moreover, the NLRP3-specific inhibitor MCC950 inhibited osteoclast differentiation both *in vitro* and *in vivo* and alleviated bone resorption. The results suggest that NLRP3 may promote osteoclastogenesis and drives inflammatory bone destruction in periodontitis, providing experimental support for NLRP3-targeted interventions (37).

### 4.3 Pyroptosis-related markers as candidate diagnostic biomarkers for periodontitis

In periodontitis-related tissues and biofluids, pyroptosis-associated mediators such as GSDMD, NLRP3, caspase-1, and IL-1β are frequently elevated and correlate with local inflammatory burden. Similar patterns have been reported in experimental periodontitis models, together with alveolar bone loss and inflammatory cell infiltration (33, 38–42). Shahbeik et al. longitudinally quantified inflammasome-related molecules in gingival crevicular fluid (GCF) before and after periodontal therapy and found that NLRP3 and IL-18 levels decreased after nonsurgical treatment (38). In severe periodontitis, GCF levels of NLRP3, caspase-1, IL-1β, and IL-18 also declined after treatment and were associated with clinical improvement, supporting their biological relevance as candidate diagnostic markers (42). Kabacaoğlu et al. assessed treatment-associated changes in pyroptosis-related indicators across saliva, serum, and GCF, providing a rationale for multi-biofluid monitoring (39). In addition, longitudinal studies suggest that GCF biomarkers change dynamically during disease progression and in response to treatment, supporting their potential utility for treatment-response follow-up (40).

Although current studies support these molecules as candidate diagnostic biomarkers for assessing periodontitis activity and tracking treatment response, barriers remain to their routine use in clinical practice. First, sampling and pre-analytical workflows are not sufficiently standardized across studies. Differences in collection time points, identification and control of biofluid contamination, and normalization strategies can reduce comparability and reproducibility. Second, cross-platform analytical validation remains limited. Assays have not been fully harmonized with respect to limits of detection, reproducibility, and inter-method agreement, and within- and between-study variability is not consistently quantified or controlled. Clinically anchored cutoffs and decision rules are still lacking. Consequently, these molecules are best viewed as translationally promising candidates for risk stratification, disease activity assessment, and longitudinal follow-up of treatment response. Their use as real-time clinical monitoring tools or to inform individualized dose adjustments will likely require standardized protocols, prospective and/or multicenter longitudinal cohorts, and external validation in independent datasets. Such studies should also test whether these markers provide incremental clinical value beyond conventional measures, including probing depth and clinical attachment loss, to facilitate their transition from experimental readouts to validated and translatable diagnostic and monitoring biomarkers (33, 38–42).

### 4.4 Pyroptosis can promote periodontitis and periodontal tissue destruction

Periodontal ligament stem cells (PDLSCs) are a key cellular source for periodontal regeneration. In periodontitis, PDLSCs can undergo GSDMD-mediated pyroptosis and release IL-1β, and IL-1β levels in GCF are positively correlated with disease severity. Pyroptosis compromises the regenerative capacity of PDLSCs and promotes the release of pro-inflammatory mediators, which can exacerbate surrounding tissue injury (23). It can also impair osteogenic differentiation and compromise periodontal repair and regeneration (41). Pyroptosis markers are expressed at significantly higher levels in periodontal tissues from patients with periodontitis than in healthy tissues, and are positively correlated with clinical inflammatory parameters, suggesting that increased pyroptosis is associated with periodontitis onset and progression (43–45). Xu et al. proposed that periodontal pathogens and their metabolites can trigger GSDMD pore formation via the canonical and non-canonical pathways, which amplifies inflammation and periodontal tissue destruction (46). Macrophages are essential immune cells. In periodontitis lesions, periodontal pathogens and inflammatory mediators can markedly disrupt macrophage metabolism and functional homeostasis, promoting pyroptosis and amplifying the release of pro-inflammatory cytokines, ultimately compromising periodontal tissue homeostasis (47). In contrast, hPDLSC-derived extracellular vesicles (EVs) can mitigate periodontal tissue injury by suppressing macrophage pyroptosis (48), supporting pyroptosis as a key driver of immune dysregulation in periodontitis. Pyroptosis can also drive the chronic progression of periodontitis and periodontal tissue destruction through multiple mechanisms, including the release of pro-inflammatory mediators, the propagation of danger signals, and the loss of cellular function (49).

**Figure 2:**
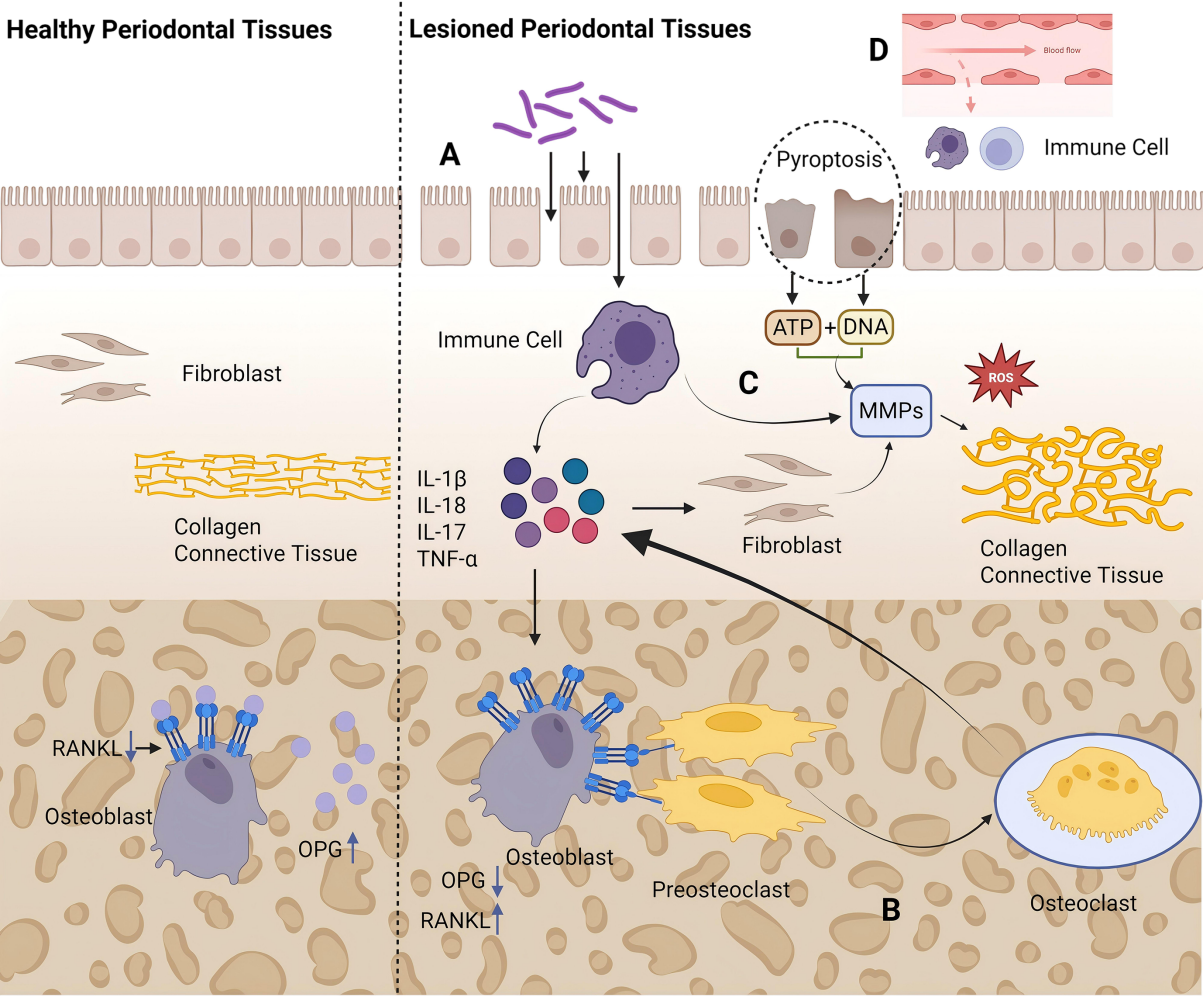
Schematic illustration of how pyroptosis drives periodontitis onset and progression. **(A)** Periodontal pathogens invade the gingival epithelium and induce immune cells to produce pro-inflammatory cytokines, such as IL-1β and TNF-α. **(B)** Pro-inflammatory cytokines stimulate osteoblasts and fibroblasts to upregulate RANKL and suppress OPG, thereby promoting osteoclast differentiation and exacerbating alveolar bone resorption. **(C)** Under inflammatory stimulation, immune cells release matrix metalloproteinases (MMPs) and ROS, which degrade the extracellular matrix of the periodontal ligament and gingival connective tissue. **(D)** Enhanced microvascular leakiness facilitates the influx of inflammatory mediators and immune cells into the tissue, leading to local inflammatory responses. This diagram was created with BioRender.com.

Pyroptosis promotes the robust release of IL-1β and IL-18, thereby amplifying the pro-inflammatory and osteoclastogenic signaling described in processes A and B. Meanwhile, lytic products from pyroptotic cells, such as ATP and DNA, act as damage-associated molecular patterns (DAMPs) that further enhance immune cell activity in process C. This creates a self-amplifying inflammatory loop and exacerbates periodontal tissue destruction through multiple routes (**Figure 2**).

## 5 Pyroptosis-targeted therapeutic strategies for periodontitis

### 5.1 Therapeutic strategies targeting GSDMD

#### 5.1.1 ATP-responsive Mg/Zn-MOF nanomaterials

This material is an ATP-responsive bimetallic metal–organic framework (Mg/Zn-MOF) developed in recent work. It can selectively sense elevated ATP signals in the periodontitis microenvironment and suppress pyroptosis by controllably releasing Zn²^+^ and Mg²^+^ (50). Owing to its rapid ATP responsiveness, this nanocomposite can promote the rapid release of stored ions toward inflamed sites with higher ATP levels, improving ion utilization and reducing diffusion-related side effects (51). The study showed that Mg(20%)/Zn-MOF maintained favorable cytocompatibility at concentrations ≤30 μg/mL, whereas cell viability declined markedly at 40 μg/mL, suggesting that higher doses may fall within a material-associated cytotoxicity risk range (50). Mg/Zn-MOF may modulate GSDMD expression through the NLRP3/caspase-1/GSDMD and caspase-11/GSDMD pathways and inhibit plasma membrane pore formation. This effect can reduce pyroptosis in periodontal cells and alleviate periodontal tissue destruction, which limits pyroptosis-driven inflammation and suggests therapeutic potential in periodontitis (50, 52). Importantly, the current evidence for this strategy is derived mainly from *in vitro* experiments and animal models, and its clinical translatability will require further validation in multi-species biofilm-host systems that better recapitulate the human oral microenvironment, followed by well-designed clinical studies. Accordingly, future work should adopt a dose-escalation approach that targets a moderate reduction in key pyroptosis readouts while also monitoring anti-infective endpoints, such as pathogen burden or biofilm biomass, to define an appropriate efficacy range and safety limits without worsening tissue damage.

#### 5.1.2 Disulfiram

Disulfiram (DSF) can covalently modify a critical cysteine residue in GSDMD and block the pore-forming activity of GSDMD-N without affecting upstream proteolytic processing. As a result, it markedly suppresses pyroptosis and reduces the release of pro-inflammatory mediators. *In vivo*, DSF improves survival in LPS-induced septic mice, further supporting its anti-inflammatory activity and translational potential. This evidence provides a pharmacological rationale for developing DSF as a GSDMD inhibitor for local oral therapy (53). However, the periodontal pocket represents a distinct microenvironment that is moist, protease-rich, and enriched in inflammatory mediators. Local delivery therefore faces rapid clearance driven by salivary washout and continuous gingival crevicular fluid exudation, and it may also entail systemic exposure through swallowing and mucosal absorption. An ideal delivery system should increase effective local exposure at the lesion site while minimizing nonproductive systemic exposure to improve the safety margin. Injectable hydrogels can help meet these dual goals.

By forming an *in situ* drug depot within the periodontal pocket or an inflamed wound bed, hydrogels enhance local retention via adhesion and spatial confinement, limiting early loss from washout and reducing peak-to-trough fluctuations associated with frequent re-dosing. This enables a more stable concentration–time profile and sustained effective exposure (54). In addition, controlled release can better match local drug input to clearance, promoting sustained perilesional delivery with uptake or consumption by local cells, while limiting diffusion to distal tissues and the circulation. This approach aligns with the exposure-safety trade-offs emphasized in disulfiram repurposing studies. Importantly, the hydrogel matrix in a wet setting may improve drug stability and microenvironmental compatibility, reduce loss of activity due to dilution, degradation, or nonspecific binding, and enable comparable or superior local anti-inflammatory efficacy at lower doses (54, 55). An injectable sodium alginate hydrogel loaded with DSF can markedly reduce inflammatory-phase markers and dampen pyroptosis-related pathway activity in neutrophils. It also promotes macrophage polarization toward a repair-associated M2 phenotype, improves endothelial function and angiogenesis, and accelerates wound closure while enhancing tissue remodeling (54). Although these findings were not generated in clinical studies of periodontitis, the ability to achieve stable local delivery and to dampen inflammatory amplification loops in a moist inflammatory setting supports hydrogels as a promising platform for drug repurposing and biomaterial-based delivery. Taken together, a more prudent translational strategy is to prioritize local, sustained-release systems that inhibit GSDMD pore formation while minimizing systemic exposure. In the oral setting, further evaluation of hydrogel residence time, maintenance of effective local concentrations, and potential systemic exposure will be essential to define the therapeutic window and facilitate clinical translation.

#### 5.1.3 Necrosulfonamide

Necrosulfonamide (NSA), a cysteine-reactive electrophilic small molecule, is a chemical inhibitor that directly targets the pore-forming step of pyroptosis. It covalently binds a critical cysteine residue in GSDMD (human Cys191; mouse Cys192) and blocks post-cleavage GSDMD-N oligomerization and plasma membrane pore formation. Consequently, NSA suppresses pyroptosis and reduces the release of pro-inflammatory cytokines such as IL-1β (56, 57). Rathkey et al. showed that NSA inhibited pyroptotic pore formation and cell lysis in murine macrophage systems and, in a dose-dependent manner, suppressed pyroptotic pore formation and cell death in human THP-1 monocytes, supporting mechanistic relevance in human cells. In the same study, the authors first established a maximum non-toxic dose of 20 mg/kg and then administered NSA at 20 mg/kg in an LPS-induced sepsis mouse model, which reduced inflammatory mediators and improved survival (56). However, its efficacy and safety in the human periodontal niche—characterized by multispecies biofilms and pronounced immune heterogeneity—remain to be validated, which is essential for a more objective assessment of its translational potential (56, 57).

#### 5.1.4 Specific antibodies

Nanobodies targeting human GSDMD offer a highly specific biologic approach to directly block the terminal pore-forming step of pyroptosis. Kopp et al. reported multiple anti-GSDMD nanobodies that inhibit GSDMD pore formation *in vitro*; inhibitory nanobodies bind the GSDMD N-terminus and sterically interfere with the oligomerization interface, preventing pore assembly without affecting upstream inflammatory caspase-mediated proteolytic cleavage (58). Schiffelers et al. further showed that antagonistic nanobodies can also suppress pyroptosis when applied extracellularly, suggesting that nanobodies may access the cytosol through nascent pores and limit subsequent pore-forming events, which supports their feasibility for exogenous administration and potential therapeutic application (59). Future studies should validate their ability to attenuate inflammatory amplification and tissue damage in systems that more closely recapitulate the human periodontal microenvironment, and should assess safety and feasibility in conjunction with local delivery strategies and pharmacokinetic-pharmacodynamic profiling.

### 5.2 Therapeutic strategies targeting the NLRP3 inflammasome

#### 5.2.1 X-box binding protein 1

X-box binding protein 1 (XBP1) is a central transcription factor of the endoplasmic reticulum (ER) unfolded protein response (UPR). It is aberrantly upregulated in tissues from patients with periodontitis and can trigger GSDMD-mediated pyroptosis by activating the NLRP3 inflammasome. Kang et al. observed marked upregulation of XBP1 in periodontitis models. Silencing XBP1 reduced pyroptosis and inflammatory cytokine expression, restored immune homeostasis, and significantly alleviated bone resorption and periodontal tissue destruction (60). XBP1s is the spliced, active form of XBP1 generated by IRE1α-mediated mRNA splicing and has strong transcriptional activity. Studies have shown that spliced XBP1 can directly bind to and enhance NLRP3 promoter activity (61). Under conditions of enhanced ER–mitochondria interactions with increased Ca²^+^ and ROS, XBP1s promotes caspase-1 activation, IL-1β maturation, and pyroptosis. Inhibition of the IRE1α–XBP1 axis can reduce NLRP3 transcription and mitigate mitochondrial injury and inflammatory responses. Zhang et al. reported that diabetes-associated periodontitis is accompanied by upregulation of IRE1α/XBP1, an M1-skewed macrophage profile, and inflammasome activation. Inhibiting this axis reduced XBP1s and ER stress markers, suppressed the NLRP3 pathway and inflammatory cytokines, and markedly alleviated bone resorption and periodontal inflammation, implicating UPR–XBP1–driven inflammasome activation in periodontal pathology (62).

A related study isolated and characterized PDLSC-Exos enriched in miR-205-5p and demonstrated that miR-205-5p directly targets and downregulates XBP1. *In vitro*, miR-205-5p reduced IL-1β, IL-18, and pyroptosis-related markers and promoted macrophage polarization from M1 to M2. *In vivo*, it alleviated gingival inflammation, improved Th17/Treg imbalance, and significantly reduced alveolar bone resorption (63). Knockdown of miR-205-5p or overexpression of XBP1 attenuated these effects, indicating that exosome-delivered miRNA therapy targeting XBP1 can reshape immune homeostasis and holds therapeutic potential for periodontitis. However, Cui et al. reported that increasing XBP1s promoted periodontal ligament cell proliferation and osteogenic differentiation, whereas inhibiting XBP1s produced the opposite effects. They proposed that moderate activation of XBP1s may be a strategy to enhance periodontal repair and regenerative potential (64). Collectively, XBP1 may exert context-dependent effects under different conditions. Future studies should clarify the dynamic balance between XBP1 activity and inflammatory signaling, define a therapeutic safety window, and minimize excessive modulation to guide clinical translation.

#### 5.2.2 MCC950

MCC950 is a selective inhibitor of NLRP3. By binding to the NACHT domain of NLRP3 and blocking its ATPase activity, MCC950 inhibits inflammasome assembly and has shown therapeutic benefits across multiple inflammatory diseases, including experimental models of periodontitis (37, 65–67). A hyperglycemic milieu can impair macrophage autophagy and promote mitochondrial ROS accumulation, activating the NLRP3 inflammasome and exacerbating periodontal inflammation and alveolar bone loss. Treatment with the NLRP3 inhibitor MCC950 can suppress macrophage pyroptosis and reduce bone resorption (35). *Pasteurella multocida* toxin markedly enhances NLRP3 signaling, promotes IL-1β release and GSDMD cleavage, and aggravates alveolar bone loss. MCC950 effectively attenuates these responses and reduces inflammation and inflammatory bone destruction (68).

Dual-crosslinked hydrogels are injectable, adhesive, multifunctional platforms for local delivery of MCC950, featuring stimulus responsiveness and sustained-release properties. *In vitro*, they can suppress the macrophage NLRP3–caspase-1–GSDMD axis and reduce IL-1β and IL-18. In periodontal cells, they can also alleviate inflammation-induced inhibition of osteogenesis. In a ligature-induced rat periodontitis model, the hydrogel markedly prolonged local retention of MCC950 compared with free drug, reduced inflammatory infiltration and collagen degradation, decreased alveolar bone resorption, and promoted bone regeneration, with good tissue compatibility. This platform offers a feasible route for precision delivery and clinical translation of NLRP3 inhibitors in periodontitis (69).

MCC950-loaded PEG–PLGA nanocarriers can markedly downregulate the NLRP3–caspase-1–IL-1β/IL-18 axis in M1 macrophages and promote resolution of inflammation. In a rat model of chronic periodontitis, this targeted nanoformulation more effectively alleviated periodontal inflammation and alveolar bone resorption than free drug. Glucose transporter 1 (GLUT1) mediates glucose uptake from the extracellular space into cells to fuel glycolysis. A study showed that GLUT1-targeted delivery to M1 macrophages increased effective lesion-site exposure to MCC950 and enhanced its anti-inflammatory and tissue-protective effects (70).

MCC950 effectively blocks NLRP3-driven IL-1β and IL-18 production and improves inflammatory outcomes across multiple models, providing a mechanistic rationale for targeting NLRP3-driven inflammation in periodontitis. However, clinical evaluation in humans is limited, and concerns regarding hepatotoxicity and pharmacokinetic stability remain major barriers to clinical translation (71, 72). Progress will require a more reproducible and predictable balance between metabolic and toxicologic liabilities and *in vivo* exposure control, alongside additional target-engagement validation in the intended patient population. In parallel, biomarker frameworks that link pathway inhibition to clinical benefit are needed to support dose selection and response monitoring. Stratification by clinical phenotype and immunopathologic endotypes should be used to reduce efficacy uncertainty driven by population heterogeneity and to define pharmacokinetic–pharmacodynamic relationships and exposure–response windows. Systematic safety assessments and actionable dose-finding strategies, with careful evaluation of the long-term risk mitigation, are essential to enable robust and testable designs for prospective clinical studies.

#### 5.2.3 Dapansutrile

Dapansutrile (OLT1177) is an orally available, small-molecule, selective NLRP3 inflammasome inhibitor. By suppressing NLRP3 activation, it reduces downstream caspase-1 activation and IL-1β/IL-18 release, attenuating inflammation implicated in a range of diseases. In a short-term study in healthy volunteers, 1,000 mg daily for 8 days was not associated with apparent adverse events or biochemical/hematological abnormalities (73). In murine models of acute arthritis, oral dapansutrile improved joint inflammatory outcomes in a dose-dependent manner (74). In an open-label, dose-adaptive, proof-of-concept phase 2a trial in acute gout flares, dapansutrile was evaluated at 100–2,000 mg/day for 8 days, with overall acceptable safety and signals of analgesic and anti-inflammatory benefit (75). Consequently, dapansutrile has emerged as a representative clinical candidate in the post-MCC950 era. Building on the dose ranges tested in humans, early translational studies in periodontitis should prioritize short-course, dose-escalation range-finding designs to iteratively define a plausible efficacy window, enabling an objective assessment of translational potential for clinical use.

#### 5.2.4 A20

A20 (*TNFAIP3*) is a canonical negative regulator of inflammation and a ubiquitin-editing enzyme with both deubiquitinase and E3 ligase activities. Downstream of receptors such as TLR/IL-1R and TNF receptors, it regulates K63- and K48-linked ubiquitin chains on key signaling molecules to rapidly terminate pro-inflammatory pathways including NF-κB, MAPK, and IRF. Under certain conditions, A20 can also restrain programmed cell death and inflammasome activation and maintain immune and tissue homeostasis (76, 77). In human gingival keratinocytes, A20 upregulation markedly reduced IL-6 and IL-8 release following *P. gingivalis* stimulation and decreased susceptibility to cell death, supporting a protective role for A20 in epithelial barrier stress responses (78). A20 overexpression reduced alveolar bone resorption, suppressed M1 polarization, and promoted an M2 phenotype, while downregulating the NLRP3–caspase-1–GSDMD axis and IL-1β/IL-18 signaling. In contrast, A20 deficiency or downregulation produced opposite effects (79). A20 can also promote ubiquitination and degradation of NEK7 through protein–protein interactions, weakening NEK7–NLRP3 binding and limiting inflammasome activation and assembly. This mechanism highlights an actionable node for therapeutic development (80). Inflammatory stimulation is associated with A20 downregulation and impaired autophagy. Restoring A20 expression can recover autophagic flux, inhibit caspase-1 activation and GSDMD cleavage, reduce pro-inflammatory mediators, and decrease pyroptosis (81, 82). A20 upregulation also reduces TRAF6 signaling, limits RANKL upregulation, suppresses osteoclast-related transcriptional programs, and attenuates periodontal ligament cells-mediated osteoclastogenesis (81). Together, A20-linked autophagy represents a potential therapeutic strategy to curb inflammatory amplification. Given that A20 is a ubiquitin-editing enzyme broadly involved in immune homeostasis, systemic upregulation of A20 may carry theoretical risks, including excessive immunosuppression, increased susceptibility to infection, or compromised tumor immune surveillance. Therefore, a more feasible translational strategy would focus on periodontal site-restricted, controllable, and reversible targeted delivery. For example, short-course interventions delivered via hydrogels, nanocarriers, or local sustained-release platforms could maintain therapeutically relevant concentrations at the lesion site while minimizing systemic exposure. Local pharmacodynamic readouts could be used to track on-target activity, potentially supporting clinical translation.

### 5.3 Therapeutic strategies targeting caspases

#### 5.3.1 Caspase-1 inhibitors

VX-765 (Belnacasan) is an orally available, small-molecule prodrug that selectively inhibits caspase-1 and also shows activity against caspase-4. It blocks IL-1β and IL-18 maturation and release, suppressing inflammasome signaling and pyroptosis. *In vivo*, VX-765 is converted by esterases to the active metabolite VRT-043198, offering favorable drug-like properties and convenient administration, and it has advanced into clinical development (83). In mouse periodontitis models, VX-765 markedly reduced inflammatory and bone-resorption markers, supporting its potential to limit periodontal bone destruction (22, 84).

Ac-YVAD-cmk is a classic peptide-based, irreversible caspase-1 inhibitor. It inhibits caspase-1 *in vitro* and *in vivo*, reduces IL-1β/IL-18 secretion and pyroptosis-related readouts, and has been used as a validation and tool compound in periodontal cell and infection models (22, 45, 85, 86). However, compared with VX-765, Ac-YVAD-cmk has less favorable druggability, selectivity, and dosing practicality and is more commonly used as pharmacological evidence in experimental settings.

#### 5.3.2 Caspase-4/5 (human)/caspase-11 (mouse) inhibitors

Z-LEVD-fmk and Ac-LEVD-cmk are cell-permeable inhibitors targeting caspase-4 and caspase-11. In human PDLSCs and periodontitis specimens, bacteria and cytosolic LPS have been shown to trigger caspase-4/GSDMD-dependent pyroptosis with concomitant IL-1β release (22). The surface protein Td92 from the periodontal spirochete *T. denticola* can activate caspase-4 via neutrophil cathepsin G and trigger pyroptosis, underscoring the feasibility of targeting caspase-4 to interrupt pathogen–host inflammatory amplification (87). Pyroptotic supernatants can inhibit osteogenesis and promote osteoclastogenesis. *In vitro* and *in vivo*, caspase-4 inhibitors (e.g., Z-LEVD-fmk) reduced GSDMD cleavage and IL-1β release, suppressed cell death and inflammation, and alleviated alveolar bone loss (22, 87–89). In clinical periodontitis samples and animal models, caspase-4/11 activity and expression are increased. Pharmacologic inhibition with Z-LEVD-fmk or Ac-LEVD-cmk, or genetic loss of caspase-11, reduced inflammatory cytokine release and alveolar bone resorption (87, 90, 91), indicating that targeting the non-canonical pyroptosis pathway may benefit periodontitis prevention and treatment.

#### 5.3.3 Pan-caspase inhibitors

Z-VAD-fmk is a cell-permeable, irreversible pan-caspase inhibitor. Across gingival fibroblasts, oral epithelial cells, and macrophages, it markedly reduces the expression of inflammatory mediators such as IL-1β/IL-6, CXCL8/10, and CCL2/5, suggesting broad suppressive effects in periodontitis-associated microenvironments (92, 93). However, systemic caspase inhibition is not uniformly beneficial. In endotoxemic shock models, Z-VAD-fmk can induce macrophage necroptosis and promote immunosuppression, which attenuates excessive inflammation and reduces mortality (92). In macrophages activated via TLR3/4, Z-VAD-fmk treatment can also shift cell death toward necroptosis and autophagy-associated death (94). Z-VAD-fmk may also indirectly induce autophagy by perturbing proteostasis pathways involving NGLY1 (95). Its effects are highly cell context–dependent; for example, in the murine macrophage-like RAW264.7 cell line, Z-VAD-fmk can enhance cell death rather than confer protection (96–98). Overall, Z-VAD-fmk may have value for modulating periodontitis-associated inflammation, but its impact on cell death varies with cell type, stimulus, and inflammatory intensity and can redirect the dominant death program from one pathway to another. Accordingly, Z-VAD-fmk is better considered as an exploratory candidate for local, short-course administration rather than systemic clinical therapy. In practice, combining it with the mechanical debridement of non-surgical periodontal therapy (NSPT) may help reduce inflammasome burden (42).

### 5.4 Natural compounds

Natural compounds are widely available and structurally diverse, and they often exhibit multi-target activity, relatively low toxicity, and favorable biocompatibility. These features have highlighted their therapeutic promise in periodontitis and have made them a growing focus of research. A range of natural bioactive constituents, including flavonoids, polyphenols, and terpenoids, have been shown to modulate key molecular nodes of pyroptosis and to attenuate periodontal inflammation and tissue destruction by regulating oxidative stress, autophagic flux, and upstream signaling pathways such as NF-κB. **Tables 1**–**3** summarize recent advances in the application of diverse natural compounds for periodontitis therapy.

**Table 1:** Polyphenolic flavonoids for the treatment of periodontitis.

Compound and molecular formula	Category	Mechanism and applications
Quercetin; C_15_H_10_O_7_	flavonol subclass	Downregulates TLR2/MyD88/NF-κB and ROS/AMPK; inhibits NLRP3 activation, caspase-1 cleavage, and pore formation (99). Reduces NLRP3-related proteins and alleviates inhibition of osteogenesis; siRNA-mediated silencing of NLRP3 mimics the effects of quercetin (100, 101).
Isorhamnetin, 3′-Methoxyquercetin; C_16_H_12_O_7_	methylated flavonol	Activates Nrf2; reduces pro-inflammatory mediators and inflammasome priming (102). Selectively inhibits the NLRP3 and AIM2 inflammasomes; decreases IL-1β and IL-18 secretion; inhibits caspase-1 activity (103). Downregulates NF-κB, NLRP3, and cleaved caspase-1; improves histopathology and inflammatory indices (104).
Nobiletin, NOB; C_21_H_22_O_8_	polymethoxyflavone	Attenuates inflammasome priming with reduced p-NF-κB/MAPKs/Akt, lowering pyroptosis susceptibility (105, 106). Upregulates SIRT1; inhibits NLRP3–caspase-1; reduces inflammatory mediators (107). Activates AMPK to enhance autophagic flux and limit ROS, reducing pore formation and lysis (108). Modulates crosstalk between autophagy and ROS, altering the expression of pyroptosis-related molecules (109).
Isoliquiritigenin, ISL; C_15_H_12_O_4_	chalcone-type flavonoid	Suppresses pyroptosis in gingival fibroblasts; attenuates NF-κB; downregulates NLRP3 and caspase-1; reduces GSDMD-N, IL-1β, and pore formation (110). Functional NLRP3 inhibitor (111). Inhibits IKKβ-NF-κB, reducing pro-inflammatory mediators and periodontal bone resorption (112).
Baicalin; C_21_H_18_O_11_	flavone glucuronide	Modulates autophagy and inflammation; downregulates pro-inflammatory mediators and PDLC inflammatory injury (113). Regulates AMPK; inhibits NLRP3; reduces IL-1β maturation (114, 115). Inhibits NF-κB; decreases ROS and inflammatory effector signaling (116). Inhibits caspase-11 non-canonical inflammasome activation; reduces pyroptosis-associated mediators (117).

**Table 2:** Isoquinoline alkaloids for the treatment of periodontitis.

Compound and molecular formula	Category	Mechanism and applications
Coptisine; C_19_H_14_NO_4_^+^	quaternary protoberberine alkaloid	Blocks caspase-1–ASC interaction; prevents NLRP3 inflammasome assembly and activation; reduces IL-1β maturation and GSDMD cleavage (118).
Berberine; C_20_H_18_NO_4_^+^	quaternary protoberberine alkaloid	Inhibits ROS-TXNIP-NLRP3; reduces pyroptosis, periodontal inflammation, and alveolar bone loss (119, 120). Modulates gut microbiota; improves periodontal bone mass and inflammatory indices (121). Thermosensitive hydrogels and Coptis herbal formulations support local delivery and lesion-site exposure (122).
Palmatine; C_21_H_22_NO_4_^+^	quaternary protoberberine alkaloid	In particulate- and infection-driven inflammatory models, reduces NLRP3-dependent pyroptosis; improves histopathology and inflammatory markers; blunts pyroptotic responses triggered by diverse danger signals (123).
Jatrorrhizine; C_20_H_20_NO_4_^+^	quaternary protoberberine alkaloid	Downregulates MAPK and NF-κB; reduces GSDMD-N and IL-1β; enhances CD39-mediated purinergic braking to inhibit inflammasomes, with anti-inflammatory and tissue-protective potential (124–126).

**Table 3:** Other compounds for the treatment of periodontitis.

Compound and molecular formula	Category	Mechanism and applications
Ginsenoside Rg1; C_42_H_72_O_14_	dammarane-type triterpenoid saponin; protopanaxatriol, PPT-type	Inhibits Drp1-mediated mitochondrial fission; improves mitochondrial dysfunction; reduces PDLC pyroptosis; protects periodontal tissues via anti-inflammatory, antioxidant, and immunomodulatory effects (127). Suppresses macrophage pyroptosis; alleviates alveolar bone resorption and local inflammation in a periapical periodontitis (128).
Dioscin; C_45_H_72_O_16_	steroidal saponin; spirostanol saponin	Inhibits K^+^ efflux and mtROS/oxidized mtDNA signaling; blocks NLRP3 assembly and caspase-1 activation; reduces IL-1β maturation and lysis; Significantly decreases alveolar bone resorption in mouse periodontitis (129).
Kynurenic acid, KA; C_10_H_7_NO_3_	quinoline carboxylic acid; tryptophan–kynurenine pathway metabolite	Reduces Ca²^+^ mobilization and mtROS; inhibits NLRP3 assembly, caspase-1 activation, and IL-1β production, with therapeutic potential for periodontitis (130–132).

The mechanisms by which natural compounds regulate pyroptosis are complex and diverse, and these agents often act through multi-target, synergistic effects. This profile provides a rich reservoir of candidates and research directions for developing novel, effective, and low-toxicity therapies for periodontitis. Their real-world translational potential largely depends on whether adequate exposure can be achieved and sustained within the periodontal microenvironment and whether efficacy can be reproduced consistently across settings. A feasible translational approach is to position natural compounds as adjunctive interventions to conventional periodontal therapy. Their incremental benefit when combined with standard care should be further validated in higher-quality animal models and well-designed clinical studies.

### 5.5 Ion channels

#### 5.5.1 The Piezo1 channel

Piezo1 is a mechanosensitive ion channel located on the plasma membrane. It forms a large trimeric complex that converts mechanical cues (e.g., stretch, shear stress, matrix stiffness, and hydrostatic pressure) into cation influx, predominantly Ca²^+^, leading to activation of downstream signaling pathways (133–137). One study reported that inflammatory stimuli activate Piezo1, promoting Ca²^+^ influx and triggering GSDMD-mediated pyroptosis. This process impairs the osteogenic differentiation of PDLSCs and exacerbates alveolar bone resorption (41). Inhibiting Piezo1 by siRNA knockdown, genetic deletion of Piezo1 in Wnt1-positive cells, or pharmacologic blockade partially restores stem cell function and markedly reduces periodontal tissue destruction. In a mouse periodontitis model, local injection of the Piezo1 inhibitor GsMTx4 also significantly attenuated alveolar bone loss (41). Piezo1 is also a key mechanosensor and potential therapeutic target in macrophage-driven gingival injury. In periodontitis, macrophages show increased Piezo1 expression and activation, which promotes M1 polarization, elevates pro-inflammatory mediators and MMP-8/MMP-13, and suppresses type I/III collagen expression in fibroblasts via paracrine signaling. These effects accelerate collagen degradation and gingival tissue destruction, Piezo1 inhibition partially reverses these changes (138). In terms of clinical translation, Piezo1 is a mechanosensitive ion channel that links mechanical cues to inflammatory amplification. Because periodontitis lesions are largely confined to the periodontal pocket and gingival crevice, where tissues experience sustained changes in mechanical loading alongside ongoing inflammation and matrix remodeling, targeting Piezo1 could influence multiple disease-relevant processes, including pro-inflammatory immune polarization, MMP-associated matrix degradation, and impaired osteogenic capacity of PDLSCs. This mechanistic profile aligns with the self-reinforcing interplay between inflammation and tissue destruction in periodontitis. Available evidence also suggests that Piezo1 is pharmacologically tractable in key effector cells, including PDLSCs and macrophages, and is associated with alveolar bone loss and gingival collagen degradation, providing a rationale for therapeutic benefits that may extend to both hard- and soft-tissue protection (41, 138).

#### 5.5.2 Purinergic channels

Purinergic channels refer to ion channels that are activated by extracellular purines, primarily ATP and ADP (139–141). In a narrower sense, the term often refers to the P2X receptor family, which comprises ligand-gated cation channels. Upon ATP binding, membrane permeability increases and a large pore–like phenotype can develop, allowing channel opening with Na^+^/Ca²^+^ influx and K^+^ efflux to initiate intracellular signaling (139–141). In contrast, P2Y receptors are G protein-coupled receptors (GPCRs) and do not form ion channels directly. In periodontal tissues, P2X7 is the most frequently discussed purinergic receptor (142–145). Inflammatory or mechanical stimuli promote ATP release. Extracellular ATP activates P2X7, alters ion fluxes, and triggers NLRP3 inflammasome activation and caspase-1 signaling, promoting IL-1β release and amplifying periodontal inflammation and bone resorption (34, 145–148). In gingival epithelial cells, ATP stimulation can induce ROS production. P2X7 can assemble with P2X4 and pannexin-1 into a signaling complex that promotes inflammasome activation and amplifies oxidative stress. Accordingly, inhibiting P2X7 and associated large-pore pathways (e.g., pannexin-1) may reduce inflammatory mediators and mitigate tissue damage (146).

Experimentally, ATP or BzATP is commonly used to activate P2X7, whereas antagonists such as A-740003, AZ10606120, or Brilliant Blue G (BBG) are used to inhibit the pathway (145, 149, 150). In a ligature-induced rat model of periodontitis, BzATP was used for *in vitro* stimulation at 50–100 μM, whereas BBG at 10 μM was sufficient to attenuate BzATP-induced responses. *In vivo*, BzATP administered intraperitoneally at 1 mg/kg/day for 11 days exacerbated inflammation and bone resorption, whereas BBG given intraperitoneally at 45.5 mg/kg every 48 h for 11 days reduced bone loss and inflammatory readouts (145). Taken together, these preclinical data suggest that P2X7 signaling is amenable to pharmacologic modulation, while highlighting the need for periodontal context-specific validation to inform clinically relevant dosing and delivery strategies.

#### 5.5.3 The TRP channel family

**Figure 3:**
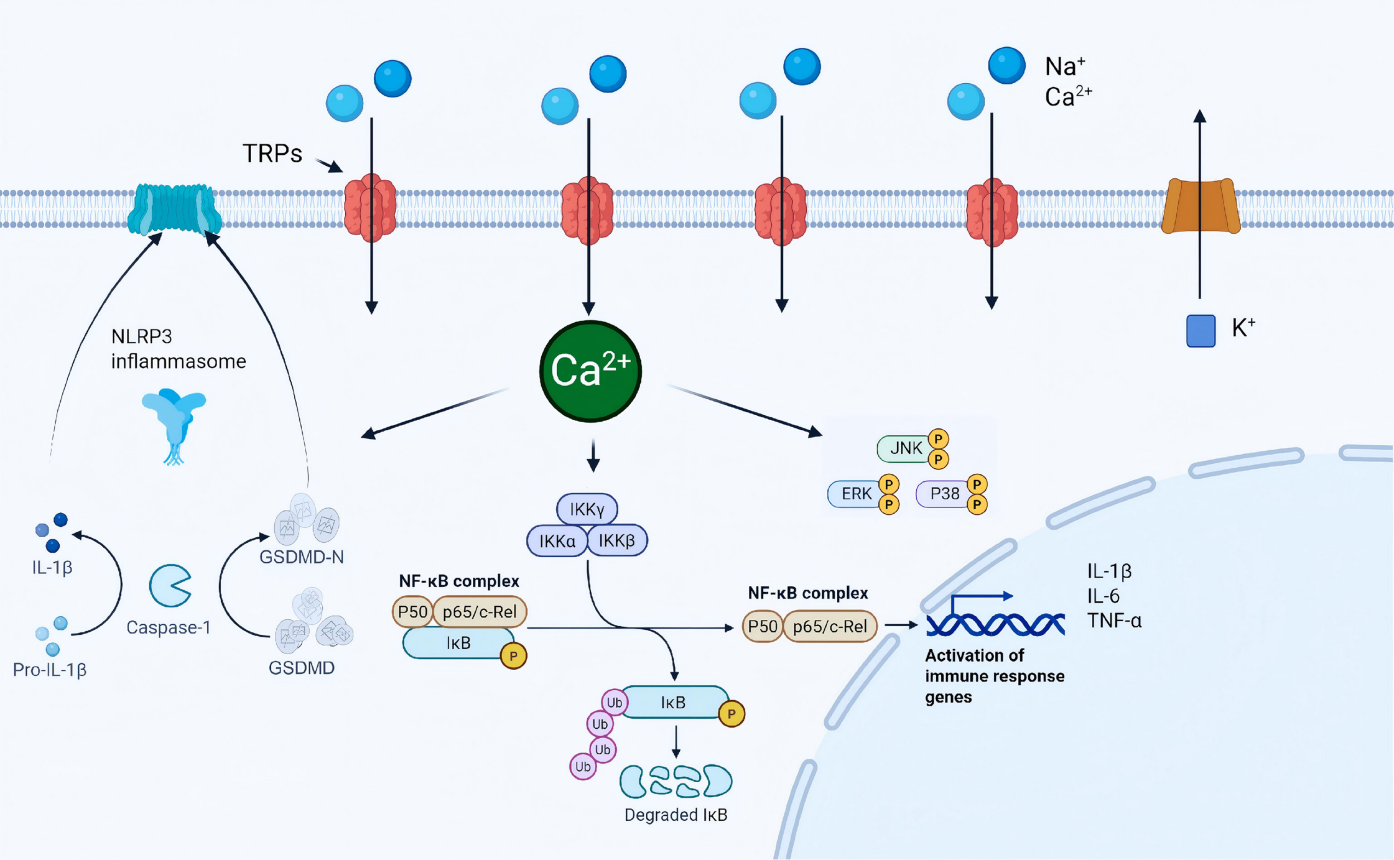
Inflammatory mechanisms of the TRP channel family. This diagram was created with BioRender.com.

The transient receptor potential (TRP) channel family comprises widely expressed, nonselective cation sensory channels located on the plasma membrane. Most TRP channels are tetrameric and respond to diverse stimuli, including heat, mechanical stretch, chemical ligands, osmolarity, pH, and redox changes (151–153). Upon activation, they primarily mediate Ca²^+^/Na^+^ influx and engage downstream signaling pathways such as NF-κB, MAPK, and inflammasomes. Major TRP subfamilies include TRPV(V1-V4), TRPA, TRPM, TRPC, TRPML and TRPP (151–153). Inflammatory mediators and mechanical loading can upregulate the expression and activity of TRPV1, TRPV4, TRPM2, and TRPA1 in cells such as PDLCs and gingival epithelial cells. This promotes Ca²^+^ influx with concomitant K^+^ efflux, enhances ROS/NF-κB signaling, and exacerbates alveolar bone resorption, supporting periodontitis progression (154–157) (**Figure 3**).

Sustained mechanical force markedly increases NLRP3, cleaved caspase-1, GSDMD-N, and IL-1β/IL-18 in periodontal ligament (PDL) progenitor cells. TRPV4 participates in this mechanotransduction process, and caspase-1 inhibition or TRPV4 blockade reduces both pyroptosis and the magnitude of bone remodeling (156). In oxidative stress–dominant microenvironments, TRPM2 can be directly activated by ROS, promoting osteoclastogenesis and bone loss. TRPM2 inhibition or knockdown decreases ROS-associated inflammatory markers and attenuates alveolar bone resorption (157). In LPS-treated PDLSCs and *in vivo* models, TRPA1 is upregulated alongside enhanced endoplasmic reticulum stress and oxidative stress. Antagonizing TRPA1 suppresses the PERK/eIF2α/ATF4/CHOP pathway and reduces cell death (154). In the periodontal ligament, temperature- and mechanosensitive channels act as hubs linking sensory and immune processes. They convert thermal and mechanical inputs into ion fluxes and inflammatory signals, amplifying a pro-inflammatory niche and shaping bone homeostasis and remodeling (155). TRPV1 is activated by capsaicin, noxious heat, protons, and endogenous lipids. Its activation promotes Na^+^/Ca²^+^ influx and membrane depolarization and increases pro-inflammatory mediators such as TNF-α and IL-1β, driving nociception and inflammatory pain signaling. Accordingly, TRPV1 represents an important target for analgesic and anti-inflammatory interventions (158, 159). TRPV1 is markedly upregulated in human periodontal tissues and in ligature-induced mouse models. Capsaicin-driven TRPV1 activation enhances STAT3 signaling and oxidative stress, elevates pro-inflammatory mediator expression, and aggravates tissue injury. In contrast, pharmacologic antagonism or genetic inhibition reverses these changes, lowers the threshold for inflammasome activation, and reduces susceptibility to pyroptosis, providing a rationale for targeting TRPV1 in periodontitis therapy (160).

The TRP channel family offers several advantages for periodontitis therapy, including lesion-site accessibility for local delivery, tiered control across pathogenic steps, and potential benefits across multiple clinical domains. First, TRP modulation may dampen mechanical and ROS-driven inflammasome activation and reduce the propensity for inflammatory cell death. This may restrain inflammatory amplification and, in turn, influence bone remodeling and alveolar bone loss (154–157). Second, it may address pain signaling, stress-associated inflammatory worsening, and oxidative stress–biased osteoclastogenic programs (154–160). Future work should define human-relevant TRP regulatory networks and leverage advanced local delivery technologies to develop subtype-selective or microenvironment-responsive modulators. Studies should map the stage- and cell-type–specific contributions of TRP subtypes across fibroblasts, immune cells, and osteoclast precursors using advanced human periodontal models and clinical specimens. They should also evaluate whether prolonged local TRP modulation affects physiological functions of periodontal tissues, including mechanosensation and reparative regeneration. Finally, optimized local platforms that balance efficacy, biosafety, and patient acceptability are needed. Such platforms could support a shift from anti-inflammatory suppression toward restoration of immune-microenvironmental homeostasis, with the goal of slowing disease progression and promoting bone regeneration.

### 5.6 Antioxidation

In periodontal lesions, pathogenic stimuli and mechanical loading can disrupt mitochondrial function and promote ROS accumulation, leading to Ca²^+^ homeostasis imbalance. Excess ROS can trigger pyroptosis, ultimately intensifying inflammation and bone resorption (161). mtROS can directly contribute to pyroptosis in cementoblasts. Scavenging or suppressing ROS markedly reduces inflammasome activity and plasma membrane pore formation, preserves cementum integrity, and supports a tissue-protective effect of targeting mtROS (161). The Nrf2/HO-1 axis, a canonical antioxidant pathway, suppresses LPS-induced pyroptosis in gingival fibroblasts. Vitamin D analogs that activate this axis reduce ROS levels and pyroptosis-associated marker expression and improve cell death outcomes and inflammatory responses (162).

N-acetylcysteine (NAC), a broad-spectrum antioxidant, activates SIRT1 and suppresses NF-κB/caspase-1 signaling, reducing pyroptosis and rescuing impaired osteogenic differentiation in human periodontal ligament fibroblasts (hPDLFs), with protective effects on periodontal tissues (163). In exogenous oxidative stress models, the lipoxin A4 derivative BML-111 activates the Nrf2/HO-1 pathway, markedly downregulates pyroptosis markers in PDLFs, and restores osteogenic potential, supporting a coordinated antioxidant and anti-pyroptotic strategy (164). Systemic enhancement of Nrf2 activity, or modulation of the KEAP1-Nrf2 axis with nutrients or small molecules, reduces oxidative stress and inflammasome activity in periodontitis models and may improve bone homeostasis through anti-pyroptotic actions (165). Additionally, the oxidative stress-sensing channel TRPM2 is upregulated during periodontitis-associated bone defects and post-extraction wound healing; TRPM2 inhibition mitigates aberrant mitochondrial dynamics, limits bone loss, and lowers susceptibility to pyroptosis, providing a rationale for multi-target interventions that integrate antioxidant, ion-channel, and inflammasome-directed approaches in periodontitis (166). Overall, antioxidant-based strategies appear feasible for incorporation into evaluation frameworks for local periodontal therapy. However, their clinical value will depend on achieving stable exposure within the real-world oral microenvironment, demonstrating reproducible efficacy with an acceptable risk profile, and establishing actionable links between local biomarker dynamics and improvements in clinical parameters. These elements are essential to delineate the appropriate indications and to support broader clinical applicability.

### 5.7 Gene regulation

Gene regulatory technologies offer multidimensional intervention strategies for periodontitis, spanning transcriptional control, epigenetic modification, non-coding RNAs, and protein-level regulation. Demethylation and RNA methylation reprogramming can directly influence the stability and translational efficiency of inflammasome-related transcripts. In conditional knockout mice lacking the RNA methyltransferase *METTL3* in periodontal mesenchymal cells, NLRP3 inflammasome activity, GSDMD cleavage, and inflammatory cytokine release were reduced, along with improved osteogenic capacity (167). Non-coding RNA–mediated post-transcriptional regulation is also critical in gingival fibroblasts(GFs) and PDLCs (168). Inhibiting the circular RNA circ_0138959 blocks LPS-induced pyroptosis in GFs, highlighting the importance of circRNA-miRNA-mRNA networks in regulating periodontal pyroptosis. Salivary exosome–derived miR-223-3p modulates NLRP3 and reduces plasma membrane pore formation, supporting the feasibility of local delivery using miRNA mimics or antisense oligonucleotides (169). Direct gene silencing (e.g., siRNA/ASO) and blockade of key signaling axes can suppress pyroptosis under combined mechanical and infectious stimuli (170). Under excessive mechanical loading, PDLCs upregulate NLRP3 through TLR4/NF-κB signaling and undergo pyroptosis. Targeting NLRP3 signaling reduces both root resorption and tissue destruction. The E3 ubiquitin ligase synoviolin suppresses inflammasome signaling and periodontitis by regulating GSDMD stability (171), underscoring the translational potential of ubiquitination-based control of proteostasis. Human periodontitis is characterized by complex polymicrobial biofilms and substantial immune heterogeneity. The clinical promise of gene-based regulatory approaches may be greatest in patients with a high inflammatory burden, marked activation of pyroptosis pathways, and suboptimal responses to standard therapy, where short-course interventions can be used to test incremental benefit. Only with feasible delivery, reversible control, local pharmacodynamic monitoring, and careful management of infection risk can these technologies move from mechanistic evidence toward a verifiable translational pathway in human populations.

### 5.8 Circadian regulation

Circadian protein BMAL1 is a key regulator of inflammatory progression, and BMAL1 together with upstream nuclear receptor pathways broadly shapes metabolic homeostasis, the intensity of immune responses, and time-of-day–dependent tissue repair. Circadian disruption can downregulate BMAL1 expression and is accompanied by heightened oxidative stress, increased cell death, and aggravated periodontitis (172). Targeting RORα to enhance circadian rhythm amplitude can indirectly strengthen upstream BMAL1 transcriptional activity and markedly attenuate alveolar bone loss and inflammation (173). Another study also showed that circadian disruption reduces BMAL1 and increases pyroptosis, culminating in increased alveolar bone resorption in mice (174). Correcting circadian disruption, or restoring BMAL1 via genetic or pharmacologic approaches, suppresses pyroptosis-related markers, dampens inflammatory responses, and mitigates bone tissue destruction (174). These observations collectively support the therapeutic potential of circadian restoration and BMAL1-targeted interventions in periodontitis.

Patients with periodontitis frequently present with circadian risk factors such as insufficient sleep, shift-work schedules, or chronic stress, suggesting that circadian status itself may influence inflammatory control and tissue repair. With direct clinical applicability in mind, two parallel directions merit pursuit. First, circadian-informed stratification metrics should be developed in clinical cohorts by linking sleep and activity patterns and peripheral circadian markers with periodontal clinical indices and local inflammatory readouts, to define candidate responder subsets and delineate the likely indication boundaries. Second, the incremental value of circadian interventions should be evaluated within standard periodontal therapy, for example through longitudinal comparisons of circadian realignment with local inflammatory control and tissue-repair outcomes, while incorporating reproducible local pharmacodynamic readouts to track whether BMAL1-related pathways are effectively restored. If these conditions are met, circadian modulation could become clinically actionable and add a new therapeutic dimension for inflammatory control and tissue protection in periodontitis (172–174).

## 6 Conclusions and perspectives

Pyroptosis plays a complex role in host defense. Its net biological impact is not linearly dictated by the absolute levels of single mediators, such as caspase-1 or IL-1β, but instead reflects an integrated, context-dependent output shaped by spatiotemporal features, the responding cell type, and the local immune microenvironment. Early in infection, timely and spatially restricted pyroptosis in infiltrating immune cells (e.g., macrophages) can promote pathogen clearance and initiate protective immune responses. Hallmarks of this protective mode include limited GSDMD pore formation and controlled IL-1 family signaling. This response is focal, transient, and self-limiting, and it does not sustain tissue injury or dysregulated bone remodeling, thereby helping preserve oral microbial homeostasis. By contrast, under persistent chronic stimulation, recurrent activation can prolong pyroptotic activity over time and extend it beyond immune cells to stromal compartments, including periodontal ligament fibroblasts and osteogenic cells, where it promotes tissue breakdown and bone destruction. This destructive pattern is characterized by sustained inflammasome activity, increased GSDMD cleavage with escalating cell death, persistently elevated pro-inflammatory mediators, and enhanced matrix degradation, which together create a feed-forward loop that reinforces alveolar bone loss and structural damage. Rather than a fixed molecular threshold separating these states, they appear to lie on a continuous functional spectrum. Consequently, a clinically actionable qualitative framework may be more appropriate than a static dose-based standard. For example, the dominant role of pyroptosis could be inferred by jointly assessing its spatial distribution (whether it remains confined to immune cells), temporal persistence (whether it tracks with phases of chronic destruction), and its coupling to tissue-damage readouts, enabling a dynamic classification of predominantly protective versus predominantly destructive activity. Periodontitis is often driven by persistent microbial challenge and chronic stimulation, a response that is initially protective can become prolonged and excessive, and its tissue-destructive consequences may outweigh antimicrobial benefits, culminating in irreversible damage. Therefore, complete pathway blockade is unlikely to be optimal. With the emergence of small-molecule inhibitors, antibody-based agents, and multi-target regimens, a central challenge is calibrated modulation that preserves essential anti-infective defense while curbing excessive pyroptosis. Additional priorities include improving target selectivity and tissue delivery, reducing the risk of immunosuppression, accounting for inter-individual heterogeneity, and establishing long-term safety.

This review summarizes the roles of pyroptosis in periodontitis pathogenesis and outlines multidimensional intervention strategies to inform stratified care and guide clinical study design, broadening directions for precision therapy in periodontitis (**Table 4**). However, most supporting evidence remains preclinical, derived largely from *in vitro* systems and rodent models, which cannot fully capture the polymicrobial biofilm architecture, diverse immune landscapes, and chronicity of human periodontitis. Accordingly, judgments about efficacy and safety should ultimately rely on subsequent human-relevant validation. The priority is not a single highly potent inhibitor, but an implementable translational pathway. Future studies could define an appropriate therapeutic window using two types of biomarkers. The first category involves pyroptosis pathway biomarkers, used to assess target tractability and inhibition strength. These include GSDMD cleavage fragments, caspase-1 activity, and levels of mature IL-1β and IL-18, which can be dynamically monitored in gingival crevicular fluid, saliva, or tissue samples. The goal is to achieve a moderate decrease from elevated levels during inflammation, observed as a clear downward trend against a high baseline, indicating effective pathway modulation, while avoiding sustained near-complete suppression at multiple time points. The precise degree of “moderation” needs to be empirically determined through longitudinal sampling across different delivery methods and patient populations. The second category includes anti-infection and barrier function markers, used to define safety boundaries and functional outcomes. These markers should be monitored in parallel with changes in pathogen load or biofilm volume, as well as signals related to innate immune defenses and mucosal barrier integrity, such as neutrophil function readouts, mucosal integrity markers, and clinical or laboratory signs indicating an increased risk of secondary infection. If pyroptosis markers decrease, but there is concomitant worsening of infection control, increased biofilm load, or exacerbated barrier dysfunction, it suggests that pathway suppression may have entered an unfavorable zone, and a decrease in inflammatory mediators should not be simply considered as “effective.” When pyroptosis-related markers decrease in alignment with stable or improved pathogen control and improved tissue repair indicators, it is more likely to be within a protective regulatory range. Conversely, when pyroptosis is strongly suppressed but accompanied by weakened infection control, deteriorated barrier function, or adverse safety signals, it is more likely to enter a destructive or high-risk zone. This framework better reflects the immuno-ecological complexity of periodontitis than a single dose threshold, and aligns with the methodological logic of immunomodulatory drug development, which anchors dosing to efficacy readouts and functional outcomes in clinical translation.

**Table 4:** Summary of therapeutic strategies for periodontitis targeting pyroptosis.

Strategies	Intervention methods	Mechanisms of action
GSDMD	Mg/Zn-MOF; DSF; NSA; Specific antibodies	ATP-responsive Mg²^+^/Zn²^+^ release; inhibits GSDMD pore formation; modulates NLRP3–caspase-1–GSDMD.
NLRP3	XBP1; MCC950; OLT1177; A20	Inhibits NLRP3 inflammasome activation; reduces caspase-1 activity; decreases GSDMD cleavage.
Caspases	VX-765; Ac-YVAD-cmk; Z-LEVD-fmk; Ac-LEVD-cmk; etc.	Inhibits caspase-1/4/5/11 activity; blocks IL-1β/IL-18 maturation and release.
Natural compounds	Quercetin; Nobiletin; Coptisine; Palmatine; Ginsenoside Rg1; etc.	Reduces pyroptosis and periodontal inflammation by modulating oxidative stress, inflammatory pathways, etc.
Ion channels	GsMTx4(Piezo1 inhibitor); A-740003; AZ10606120; BBG; Pannexin-1(P2X7 inhibitor); TRP channel family	Inhibits Ca²^+^/Na^+^, etc. influx; blocks NLRP3 inflammasome activation and downstream pyroptosis.
Antioxidation	NAC; BML-111; etc.	Modulates oxidative stress; attenuates pyroptosis and impaired osteogenic differentiation via SIRT1/NF-κB/caspase-1, Nrf2/HO-1, etc.
Gene regulation	*XBP1* silencing; *METTL3* knockout; miRNAs; synoviolin; etc.	Multi-level intervention across transcription, epigenetic modifications, and non-coding RNAs.
Circadian regulation	BMAL1	Upregulates or restores BMAL1; inhibits GSDMD-mediated pyroptosis; mitigates inflammatory responses.

From a dosing and regimen perspective, short-course, dose-escalation designs can be used to evaluate treatment effects in staged steps and to identify the lowest intervention intensity that yields clinical benefit. This approach also allows efficacy durability and potential risks to be weighed in parallel, ultimately defining a clinically acceptable therapeutic range. For chronic periodontitis that requires long-term management, local delivery with reversible modulation is often preferable. Lesions are largely confined to periodontal pockets and gingival crevices, making site-restricted dosing feasible. Local administration can limit systemic exposure and support more controllable maintenance of therapeutically relevant concentrations within the lesion, which may improve tolerability and reduce systemic risks associated with immunosuppression. Marked immune heterogeneity in periodontitis suggests that pyroptosis-targeted approaches may yield added benefit in selected subgroups, such as patients with high inflammatory burden, persistent activation of pyroptosis pathways, and suboptimal responses to standard therapy. Consequently, clinical studies should incorporate mechanism-based stratification and reproducible local pharmacodynamic monitoring so that the extent of pathway suppression can be linked to clinical benefit in a testable manner. As these elements mature, pyroptosis-targeted interventions are more likely to be integrated into combination regimens for periodontitis in a controlled and evaluable way. Owing to the scope and length constraints of this review, we may not be able to discuss all potential targets and therapeutic strategies in detail. Nevertheless, we believe that, as new agents and enabling technologies continue to emerge, more precise modulation of pyroptosis is expected to yield breakthroughs, ultimately advancing toward clinical application and providing a more compelling evidence base for inflammatory control, individualized management, and improved prognosis in periodontitis.

## Glossary

LPS: Lipopolysaccharide
GSDMD: Gasdermin D
GSDMD-N: The N-terminal domain of gasdermin D
IL-1β: Interleukin-1β
IL-18: Interleukin-18
GSDMC: Gasdermin C
GSDME: Gasdermin E
DNA: Deoxyribonucleic acid
STING: Stimulator of interferon genes
cGAMP: Cyclic GMP-AMP
ATP: Adenosine triphosphate
ROS: Reactive oxygen species
ASC: Apoptosis-associated speck-like protein containing a CARD
NLRP3: NOD-like receptor family pyrin domain-containing 3
TNF-α: Tumor necrosis factor alpha
FADD: Fas-associated death domain protein
Apaf-1: Apoptotic protease-activating factor 1
Cyt-c: Cytochrome c
pH: Potential of hydrogen
GzmA: Granzyme A
GzmB: Granzyme B
*P.Gingivalis*: *Porphyromonas gingivalis*
*T. Forsythia*: *Tannerella forsythia*
*T. Denticola*: *Treponema denticola*
NLRC4: NOD-like receptor family CARD domain-containing protein 4
PDLSCs: Periodontal ligament stem cells
GCF: Gingival crevicular fluid
RANKL: Receptor activator of NF-κB ligand
OPG: Osteoprotegerin
MMPs: Matrix metalloproteinases
DAMPs: Damage-associated molecular patterns
EVs: Extracellular vesicles
Mg/Zn-MOF: Metal–Organic Framework
DSF: Disulfiram
NSA: Necrosulfonamide
XBP1: X-box binding protein 1
ER: Endoplasmic reticulum
UPR: Unfolded protein response
IRE1α: Inositol-requiring enzyme 1 alpha
PDLSC-Exos: Periodontal ligament stem cell-derived exosomes
mRNA: Messenger ribonucleic acid
Th17: T helper 17 cells
Treg: T regulatory cells
NACHT domain: NAIP, CIITA, HET-E, and TP1
PEG–PLGA: Poly(ethylene glycol)–poly(lactic-co-glycolic acid)
GLUT1: Glucose transporter 1
OLT1177: Dapansutrile
A20: *TNFAIP3*
IL-6: Interleukin-6
IL-8: Interleukin-8
TLRs: Toll-like receptors
IL-1R: Interleukin-1 receptor
TNF: Tumor necrosis factor
K63/K48: K63/K48-linked ubiquitin chains
NF-κB: Nuclear factor kappa B
MAPK: Mitogen-activated protein kinase
IRFs: Interferon regulatory factors
NEK7: NIMA-related kinase 7
NIMA: Never in mitosis A
TRAF6: Tumor necrosis factor receptor–associated factor 6
VX-765: Belnacasan
CXCL8: C-X-C motif chemokine ligand 8, interleukin-8 (IL-8)
CXCL10: C-X-C motif chemokine ligand 10, IP-10
CCL2: C-C motif chemokine ligand 2, MCP-1
CCL5: C-C motif chemokine ligand 5, RANTES
NGLY1: N-glycanase 1
NSPT: Non-surgical periodontal therapy
MyD88: Myeloid differentiation primary response 88
AMPK: AMP-activated protein kinase
siRNA: Small interfering RNA, short interfering RNA
Nrf2: Nuclear factor erythroid 2–related factor 2
AIM2: Absent in melanoma 2
Akt: PKB, Protein kinase B
SIRT1: NAD-dependent protein deacetylase sirtuin-1
IKKβ: Inhibitor of nuclear factor kappa B kinase subunit beta
TXNIP: Thioredoxin-interacting protein
CD39: Cluster of differentiation 39
Drp1: Dynamin-related protein 1
mtROS: Mitochondrial reactive oxygen species
mtDNA: Mitochondrial DNA
ADP: Adenosine diphosphate
GPCRs: G protein-coupled receptors
BBG: Brilliant Blue G
PDLCs: Periodontal ligament cells
TRP: Transient Receptor Potential
PERK: Protein kinase R (PKR)-like endoplasmic reticulum kinase
eIF2α: Eukaryotic translation initiation factor 2 alpha
ATF4: Activating transcription factor 4
CHOP: C/EBP homologous protein
NAC: N-acetylcysteine
hPDLFs: Human periodontal ligament fibroblasts
GFs: Gingival fibroblasts
ASO: Antisense oligonucleotide
